# Identification of Allobaculum mucolyticum as a novel human intestinal mucin degrader

**DOI:** 10.1080/19490976.2021.1966278

**Published:** 2021-08-30

**Authors:** Guus H. van Muijlwijk, Guido van Mierlo, Pascal W.T.C. Jansen, Michiel Vermeulen, Nancy M.C. Bleumink-Pluym, Noah W. Palm, Jos P.M. van Putten, Marcel R. de Zoete

**Affiliations:** a Department of Medical Microbiology, University Medical Center Utrecht, Utrecht, Netherlands; b Department of Molecular Biology, Faculty of Science, Radboud Institute for Molecular Life Sciences, Oncode Institute, Radboud University Nijmegen, Nijmegen, The Netherlands; c Department of Biomolecular Health Sciences, Utrecht University, Utrecht, Netherlands; d Department of Immunobiology, Yale University School of Medicine, New Haven, CT, USA; e Laboratory of Systems Biology and Genetics, Institute of Bioengineering, School of Life Sciences, Ecole Polytechnique Fédérale de Lausanne (EPFL), LausanneCH-1015, Switzerland

**Keywords:** *Allobaculum mucolyticum*, microbiota, intestinal mucin, mucin *o-*glycans, mucin degradation, cazymes, glycosidase, pathobiont, proteome

## Abstract

The human gut microbiota plays a central role in intestinal health and disease. Yet, many of its bacterial constituents are functionally still largely unexplored. A crucial prerequisite for bacterial survival and proliferation is the creation and/or exploitation of an own niche. For many bacterial species that are linked to human disease, the inner mucus layer was found to be an important niche. *Allobaculum mucolyticum* is a newly identified, IBD-associated species that is thought be closely associated with the host epithelium. To explore how this bacterium is able to effectively colonize this niche, we screened its genome for factors that may contribute to mucosal colonization. Up to 60 genes encoding putative Carbohydrate Active Enzymes (CAZymes) were identified in the genome of *A. mucolyticum*. Mass spectrometry revealed 49 CAZymes of which 26 were significantly enriched in its secretome. Functional assays demonstrated the presence of CAZyme activity in *A. mucolyticum* conditioned medium, degradation of human mucin *O*-glycans, and utilization of liberated non-terminal monosaccharides for bacterial growth. The results support a model in which sialidases and fucosidases remove terminal *O*-glycan sugars enabling subsequent degradation and utilization of carbohydrates for *A. mucolyticum* growth. *A. mucolyticum* CAZyme secretion may thus facilitate bacterial colonization and degradation of the mucus layer and may pose an interesting target for future therapeutic intervention.

## Introduction

The intestinal microbiota plays an important role in host health and disease. Due to their close association with the host epithelium, bacteria occupying the intestinal mucosal niche are thought to have a preponderant effect on host health. For example, their close proximity to the intestinal epithelium increases the chances of activating host immune responses, which may drive excessive inflammatory responses as seen in inflammatory bowel disease (IBD).^1^

In order to provide protection and avoid excessive inflammatory responses, the intestinal epithelial cells are covered by a viscous mucus layer that protects them from direct contact with potentially harmful bacteria. This mucus layer primarily consists of heavily glycosylated mucin proteins, such as MUC2, that are highly decorated with branched *O*-glycans linked to serine/threonine-rich repeats in the polypeptide backbone. These mucin O-glycans retain large amounts of water (>90% of the mucin weight is due to water), which gives the mucus layer its lubricant properties. Furthermore, mucin glycans also protect the peptide backbone from proteolytic degradation by bacterial and host proteases, thereby safeguarding its main function as a mucosal firewall.

Many enteropathogenic bacteria have developed mechanisms to breach the mucus barrier, e.g. through flagella-driven propulsion. However, in recent years it has also become clear that several commensal bacteria, such as *Akkermansia muciniphila* and *Bacteroides thetaiotamicron*, have adopted a mucus-dwelling lifestyle and feed on the monosaccharides present in mucin *O*-glycans.^2–5^ To enable this, these bacteria employ one or more mucin *O*-glycan specific glycoside hydrolases that release the monosaccharides that make up the glycan chain. The glycoside hydrolase families required for mucin *O*-glycan degradation have previously been described by Tailford *et al*.^6^

A decreased mucus layer thickness and increased bacterial invasion into the inner mucus layer are well-known phenomena in IBD pathology.^7^ Our group has previously demonstrated that IgA-coated bacteria are inclined to encroach toward the intestinal epithelial cells and can cause intestinal inflammation in mouse models of colitis.^8^ One of these highly IgA-coated bacteria is a newly identified bacterium from the *Allobaculum* genus: *Allobaculum mucolyticum sp. nov*. Little is known about bacteria from the *Allobaculum* genus, which are part of the *Erysipelotrichaceae* family and inhabitants of the intestinal microbiota. The first reported member of this genus is *Allobaculum stercoricanis*, a Gram-positive rod shaped bacterium isolated from dog feaces.^9^ *Allobaculum* species are mainly identified on the basis of high throughput 16S rRNA gene sequencing, often in studies that focus on changes in gut microbiota composition of mice after dietary interventions. The relative abundance of *Allobaculum* species in mice and rats has been correlated with aging, high fat diets, and fatty acid metabolism.^10–16^ Besides correlations with fatty acid metabolism, Herrmann *et al*. reported *Allobaculum* to be an active glucose utilizer and producer of lactate and butyrate.^13,14^ These reports are in line with previous reports about bacteria from the *Erysipelotrichaceae* family, which are often associated with a western or high fat diet.^17,18^

*Allobaculum* species have also been implicated in playing a role in inflammatory processes. For example, Cox *et al*.^16^ reported a positive correlation between *Allobaculum* relative abundance and levels of ileal RORγT and IL-17 levels and protection from metabolic syndrome in mice. Another, more recent report by Miyauchi *et al*. causatively linked an *Allobaculum* strain (OTU002) to increased susceptibility to experimental autoimmune encephalitis.^19^ This was attributed to this bacterium’s ability to adhere to small intestinal epithelial cells which induces the expansion of inflammatory intestinal T helper 17 cells. Finally, *A. mucolyticum* was originally isolated from an ulcerative colitis patient (UC) based on its high levels of IgA coating. Mice that were colonized with this strain as part of a microbial community also developed IgA responses toward this bacterium and developed much more severe colitis upon exposure to DSS.^8^ This suggests that *A. mucolyticum* is immunogenic *in vivo* and could play an important role in the development of intestinal inflammation.

As the reports about *Allobaculum* species are increasing in number, and we and others have reported that bacteria within the *Allobaculum* genus belong to the immunogenic inhabitants of the intestinal mucosal niche, a more detailed characterization of bacteria belonging to this genus is warranted. Here, we subjected *A. mucolyticum* to a thorough genomic and functional characterization. We show that *A. mucolyticum* can secrete a wide repertoire of mucin *O*-glycan targeting carbohydrate active enzymes (CAZymes), which allow it to effectively degrade and feed on intestinal mucins. These potent mucolytic capabilities suggest it can effectively colonize and degrade the protective mucus layer of its host.

### Results

#### Allobaculum mucolyticum *utilizes both dietary- and host-derived glycans for growth*

**Figure 1.**
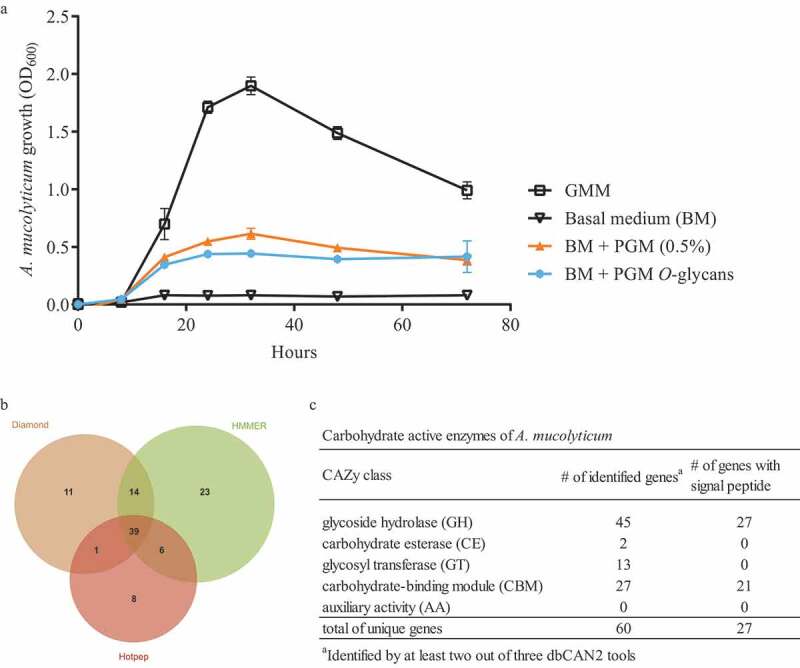
***A. mucolyticum* growth on mucin and mucin *O* glycans, and identification of carbohydrate active enzymes (CAZymes) in its genome**. (a) *A. mucolyticum* growth was assessed over a 72 h period by measuring optical density at 600 nm (OD_600_). Bacteria were grown in Gut Microbiota Medium (GMM), basal medium (BM), or basal medium supplemented with porcine gastric mucin (0.5% w/vol) or purified PGM *O-*glycans (10 mg/mL). Data represent the mean ± SD of three independent experiments. (b) Proportional Venn diagram showing the number of identified CAZyme encoding genes using the Diamond, HMMER and Hotpep tools that are part of the dbCAN2 metaserver analysis. (c) The table shows the CAZymes and the presence of signal peptides, separated by CAZyme class, that were identified by at least two out of three dbCAN2 tools

*Allobaculum* species are inhabitants of the intestine and thus encounter multiple dietary- and host-derived nutrients. To investigate the nutrient requirements of *A. mucolyticum*, we assessed its growth over a period of 72 hours in the presence of a variety of substrates. Robust growth of *A. mucolyticum* was observed in an enriched gut microbiota medium (GMM), containing multiple mono- and disaccharides, such as glucose, fructose, maltose and cellobiose (Figure 1a). To examine whether other, dietary-derived sources of glycan can also be used for growth, *A. mucolyticum* was grown in a basal medium supplemented with the plant polysaccharide inulin (Figure S1). The basal medium alone, which lacks all glycans present in GMM, did not support the growth of *A. mucolyticum*. Supplementation of basal medium with inulin resulted in firm growth, demonstrating the bacterium’s ability to utilize dietary-derived carbon sources.

As several other *Allobaculum* species have been reported to be closely associated with the intestinal mucosal niche we next investigated whether *A. mucolyticum* could also use mucins for growth. Hereto, basal medium was supplemented with porcine gastric mucin (PGM, 0.5% w/vol) and this was sufficient to support *A. mucolyticum* growth (Figure 1a). To assess whether mucin *O*-glycans and/or the mucin peptide backbone peptide promoted bacterial growth, bacteria were grown in basal medium supplemented with purified mucin *O*-glycans (10 mg/mL). After supplementation with these glycans *A. mucolyticum* was able to grow to a similar optical density as on complete porcine gastric mucin (0.5% w/vol) (Figure 1a). This showed that the *O*-glycans present on mucin are sufficient to support the growth of *A. mucolyticum* and indicates that it can, in addition to dietary-derived glycans, also use host-derived mucin as a growth substrate.

**The genome of *Allobaculum mucolyticum* predicts the presence of a wide range of *O*-glycan-targeting carbohydrate active enzymes (CAZymes)**

To investigate how *A. mucolyticum* can utilize complex *O*-glycans, we interrogated the available *A. mucolyticum* genome for the presence of carbohydrate active enzymes (CAZymes) using the dbCAN2 metaserver pipeline for automated CAZyme annotation.^20^ This pipeline uses a combination of three different annotation tools (Diamond, HMMER and Hotpep) to increase accuracy in predicting and annotating CAZymes within bacterial genomes. This analysis revealed a total of 102 putative ORFs with putative CAZy domains (Figure 1b).

**Figure 2.**
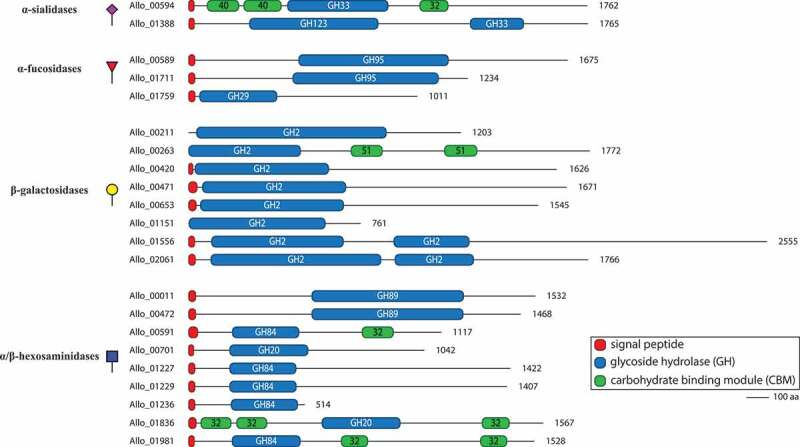
**Domain architecture of putative mucin *O-*glycan targeting CAZymes in genome of *A. mucolyticum.*** CAZymes are divided in groups based upon their predicted enzyme activity: from top to bottom, α-sialidases, α-fucosidases, β-galactosidases and α/β-hexosaminidases. The displayed domains are those identified by HMMER and SignalP 4.0 and are drawn to scale with the size of the polypeptide backbone, in number of amino acids, indicated at the right side of each protein

For increased accuracy, we discarded hits that were not identified by at least two out of three tools for further analysis. Among the remaining 60 ORFs multiple types of CAZy domains could be detected, such as glycoside hydrolase (GH), carbohydrate esterase (CE), glycosyl transferase (GT) domains, and carbohydrate-binding modules (CBM), with some genes encoding a combination of multiple domains (Figure 2c). Many of the ORFs, especially those containing glycoside hydrolase domains, also encode signal peptides, suggesting that the proteins may be transferred to the bacterial surface or secreted into the environment. The wide repertoire of secreted and non-secreted CAZymes suggests that *A. mucolyticum* is highly adapted to living in the glycan-rich mucus niche.

Closer inspection of the collection of CAZymes identified in the *A. mucolyticum* genome revealed at least 22 glycoside hydrolases that were predicted to specifically target *O*-glycans as found on mucins, of which the majority contains a signal peptide (Table 1). Among the 22 glycoside hydrolases are two sialidases (GH33) and three fucosidases (GH29/95), which target the monosaccharides often found at terminal positions of mucin *O*-glycans, and eight different galactosidases (GH2) and nine hexosaminidases (GH20/GH84/GH89), which are predicted to hydrolyze the underlying glycosidic linkages. The gene structure of the 22 putative enzymes, with the locations of the predicted glycoside hydrolase domains and additional domains (Figure 2), clearly demonstrates the great diversity in domains, domain organization and predicted protein sizes – also between genes predicted to encode the same class of enzymes. Transcription and translation of the entire repertoire of mucin-targeting CAZymes would allow degradation of the wide variety of linkages commonly found within mucin *O*-glycans.

**Table 1.** Putative mucin *O*-glycan targeting CAZymes of *A. mucolyticum.*

Specificity	Gene_ID (putative activity)	CAZy family domains^a^	Signal peptide (aa)	Protein size (aa)
Sialidase	Allo_00594 (exo-α-sialidase)	CBM40,CBM40,GH33,CBM32	Y(1-25)	1762
	Allo_01388 (exo-β-N-acetylgalactosaminidase, exo-α-sialidase)	GH123,GH33	Y(1-25)	1765
Fucosidase	Allo_00589 (exo-α-1,2-L-fucosidase)	GH95,CBM51	Y(1-29)	1675
	Allo_01711 exo-α-1,2-L-fucosidase)	GH95,CBM51	Y(1-29)	1234
	Allo_01759 (exo-α-1,3/1,4-L-fucosidase)	GH29	Y(1-30)	1011
Galactosidase	Allo_00211 (exo-β-galactosidase)	GH2,CBM51,CBM67	N	1203
	Allo_00263 (exo-β-galactosidase)	GH2,CBM51,CBM51	N	1772
	Allo_00420 (exo-β-galactosidase)	CBM32,GH2	Y(1-20)	1626
	Allo_00471 (exo-β-galactosidase)	GH2,CBM32,CBM67,CBM71	Y(1-39)	1671
	Allo_00653 (exo-β-galactosidase)	GH2,CBM32,CBM67,CBM71	Y(1-35)	1545
	Allo_01151 (exo-β-galactosidase)	GH2,CBM57	N	761
	Allo_01556 (exo-β-galactosidase)	GH2,GH2	Y(1-28)	2555
	Allo_02061 (exo-β-galactosidase)	GH2,GH2	Y(1-31)	1766
Hexosaminidase	Allo_00011 (exo-α-N-acetylglucosaminidase)	GH89,CBM32	Y(1-33)	1532
	Allo_00472 (exo-α-N-acetylglucosaminidase)	GH89	Y(1-33)	1468
	Allo_00591 (exo-β-N-acetylglucosaminidase)	GH84,CBM32	Y(1-42)	1117
	Allo_00701 (β-N-acetylglucosaminidase)	GH20	Y(1-24)	1042
	Allo_01227 (exo-β-N-acetylglucosaminidase)	GH84,CBM32	Y(1-29)	1422
	Allo_01229 (exo-β-N-acetylglucosaminidase)	GH84,CBM32	Y(1-29)	1407
	Allo_01236 (exo-β-N-acetylglucosaminidase)	GH84,CBM32	Y(1-29)	514
	Allo_01836 (β-N-acetylglucosaminidase)	CBM32,CBM32,GH20,CBM32	Y(1-35)	1567
	Allo_01981 (β-N-acetylglucosaminidase)	GH84,CBM32,CBM32	Y(1-27)	1528

^a^Identified domains are listed from N to C terminus of the peptide chain

#### A. mucolyticum *secretes a large array of mucin* O-*glycan-targeting CAZymes*

In order to establish which of the predicted mucin-targeting CAZymes is produced and secreted by *A. mucolyticum* under *in vitro* culturing conditions, both the bacterial whole-cell lysate and the conditioned medium from bacteria cultured for 24 h in complete GMM were subjected to proteomic analysis using mass spectrometry. In addition, the conditioned media from bacteria grown for 24 h in basal medium or basal medium supplemented with PGM were analyzed to investigate the effect of glycan availability on the regulation or secretion of CAZymes. Mapping of the detected peptide fragments to the *A. mucolyticum* genome revealed a total of 1005 proteins in the whole-cell lysate and the different conditioned media fractions. Since *A. mucolyticum* has a predicted 2255 genes, these results show that nearly 45% of all genes are translated under the conditions tested.

Since many CAZymes contain putative signal peptides and are predicted to be secreted, we next analyzed the total *A. mucolyticum* secretome by focusing on proteins enriched in the conditioned media compared to the whole-cell lysate. This showed that 86 proteins were significantly enriched (>10 fold) in the conditioned medium of at least one of the three different growth conditions (Figure S2). For most proteins, the highest enrichment was detected in the conditioned medium of bacteria grown in GMM. Basal medium supplemented with PGM largely mimicked the protein secretion profile of complete GMM, suggesting that PGM provides similar regulatory cues or secretion signals. Basal medium did not result in a large number of significantly enriched secreted proteins, which correlated with the poor growth in this medium.

**Figure 3.**
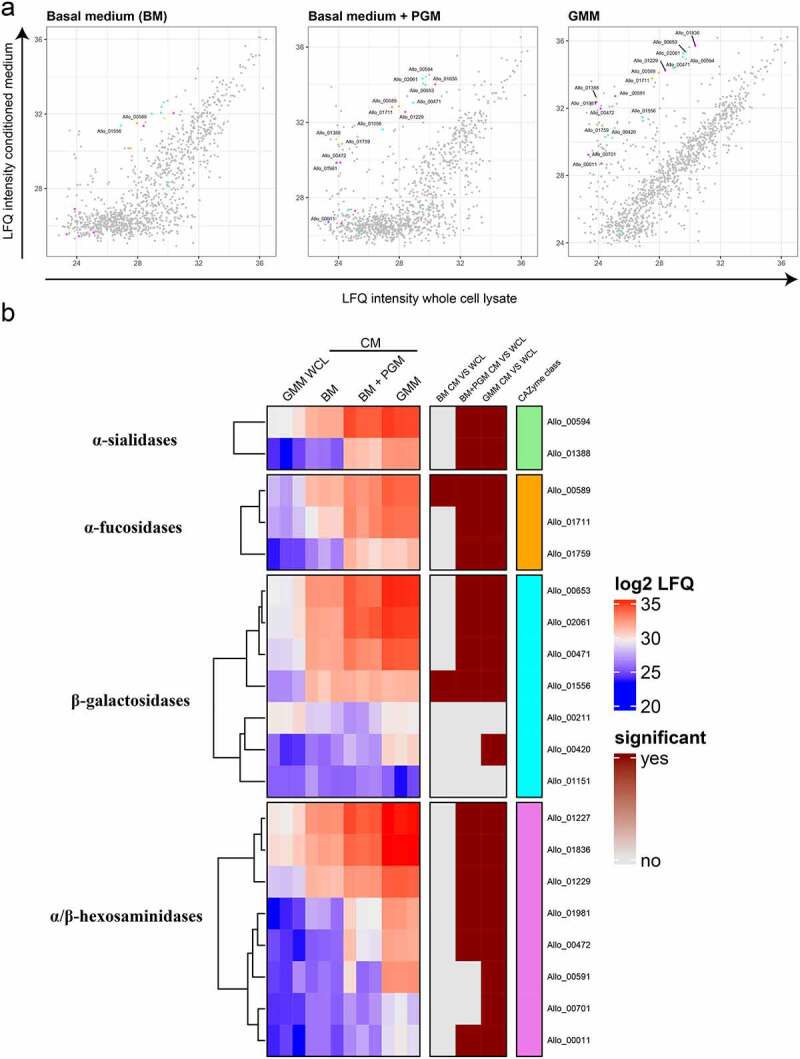
**Mass spectrometry analysis of the *A. mucolyticum* proteome demonstrates the presence of mucin *O-*glycan targeting CAZymes**. Conditioned media (CM) from 24 h cultures in basal medium (BM), basal medium + PGM and Gut Microbiota Medium (GMM) and a whole cell lysate (WCL) from the GMM culture were subjected to mass spectrometry analysis, with three biologically independent replicates per condition. (a) The scatter plots show the log2 LFQ from whole cell lysate of the GMM culture (X-axis) and the secretomes from the three different media (Y-axis). The colored dots indicate the putative *O-*glycan targeting CAZymes with their color matching the color of their putative CAZyme class shown in the heat map. (b) The red/blue heat map displays the proteins and their relative enrichment in the three different secretomes compared to the GMM WCL. Significance (depicted in the brown heatmap) was calculated using a t-test and indicates a > 10 fold enrichment in the respective secretome versus GMM WCL with an FDR < 0.05

A closer inspection of the detected proteins revealed that out of the 60 putative CAZymes identified in the genome, 49 were detected using mass spectrometry of which 26 were significantly enriched in the conditioned medium of at least one of the growth conditions (Figure S3). Eighteen of these were predicted mucin-targeting CAZymes (Figure 3), indicating that over 80% (18 out of 22) of the predicted mucin-targeting CAZymes were produced and secreted into the culture media. Strikingly, the 18 mucin-targeting CAZymes were among the most abundant proteins detected. This analysis demonstrates that *A. mucolyticum* produces and secretes a large array of putative mucin *O-*glycan targeting glycoside hydrolases which together are predicted to efficiently deglycosylate mucin proteins.

#### Functional activity of the secreted CAZymes

**Figure 4.**
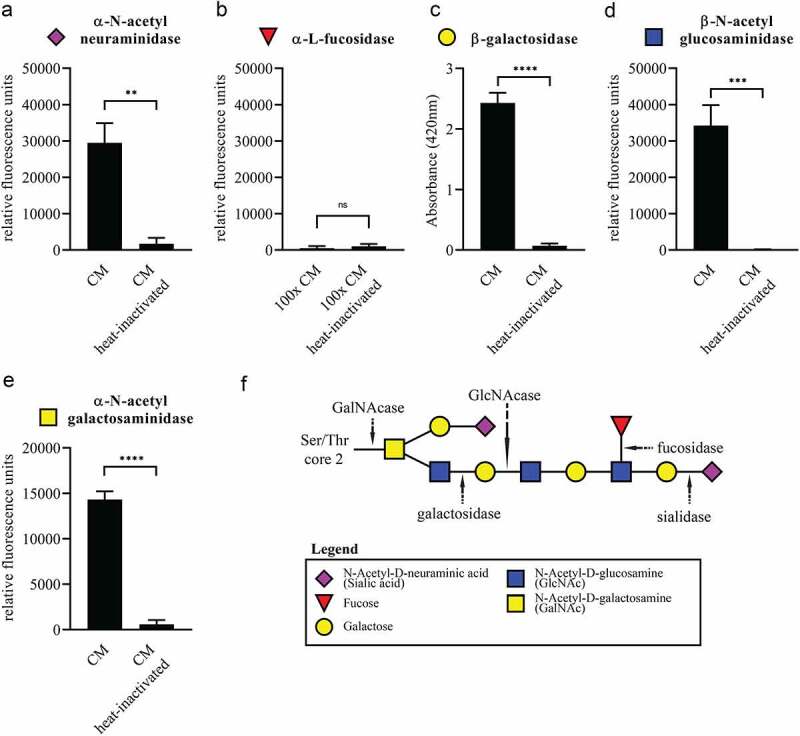
Enzymatic activity of mucin *O*-glycan-targeting CAZymes in conditioned medium of *A. mucolyticum*. Filter-sterilized conditioned medium (CM) from a 48 h culture of *A. mucolyticum* grown in Gut Microbiota Medium was incubated for two hours at 37°C with fluorescent 4-methylumbelliferone linked (a) α-N-acetylneuraminic acid (sialic acid), (b) α-fucose, (d) β-GlcNAc and (e) α-GalNAc or the chromogenic substrate (c) 2-Nitrophenyl β-D-galactopyranoside (ONPG) for the detection of enzymatic activities. A 100x concentrated conditioned medium was used with the α-fucose substrate. (f) Schematic composition of a typical core 2 mucin *O*-glycan and the target site for each enzyme class. Data represent the mean ± SD of three independent experiments. Statistical significance was determined by an unpaired t test using GraphPad Prism software. **, P < .01; ***, P < .001; ****, P < .0001; ns, not significant

**Figure 5.**
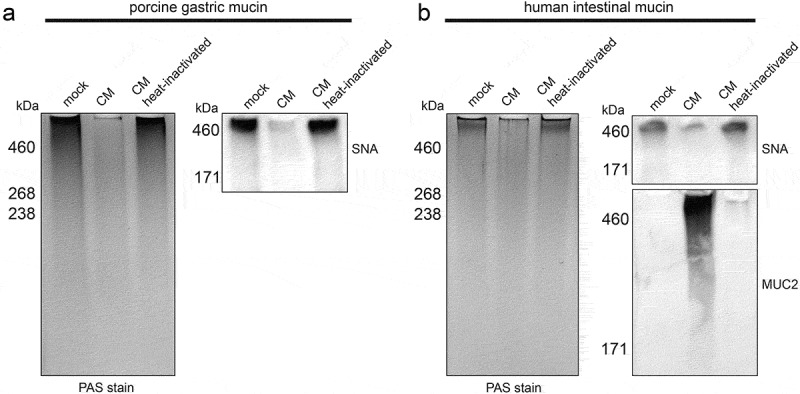
**Enzymatic activity within *A. mucolyticum* conditioned medium degrades porcine and human mucins**. (a) Porcine gastric mucin or (b) human intestinal mucin (both 10 mg/mL) were mixed 1:1 with GMM (mock) or active or heat-inactivated *A. mucolyticum* conditioned medium (CM) from a 48 h culture grown in GMM, and incubated overnight at 37°C. After separation using SDS-PAGE, mucins were either detected in-gel using a Period acid-Schiff (PAS) stain or electroblotted and detected using SNA lectin, and in the case of human intestinal mucin also with an anti-MUC2 antibody. Molecular masses are indicated in kilodaltons (kDa)

Mucin *O-*glycans, such as the typical core 2 mucin *O*-glycan depicted in figure 5f, are made up of multiple covalently linked monosaccharides. To degrade such glycans the linkages between the different monosaccharides need to be hydrolyzed by CAZymes with the correct linkage specificity. To verify whether the CAZymes secreted by *A. mucolyticum* are enzymatically active and able to hydrolyze linkages found in mucin *O*-glycans, conditioned medium or heat-inactivated conditioned medium (30 minutes at 98°C) from a 48 h culture was incubated with five different monovalent fluorescent or chromogenic substrates to detect the presence of sialidase, fucosidase, galactosidase, N-acetyl-glucosaminidase and N-acetyl-galactosaminidase activities. This resulted in clear signals for the presence of α-sialidase, β-galactosidase, β-GlcNAcase and α-GalNAcase activities in *A. mucolyticum* conditioned medium (Figure 4a-e). Although multiple fucosidases were detected in the secretome during the proteomics analysis, no fucosidase activity could be detected – even after concentrating the culture media 100x. An alternative approach using chemoselective labeling of the fucosidases with an activity-based probe, which can detect fucosidase activity in enzymes with both GH29 and GH95 glycosyl hydrolase domains, also did not detect fucosidase activity. Despite the lack of fucosidase activity, the combination of identified enzymatic activities would likely be able to effectively target and degrade *O*-linked glycans, such as found on mucins.

#### *Mucin degradation and utilization by* A. mucolyticum

To assess whether secreted CAZymes in the *A. mucolyticum* conditioned medium are sufficient to degrade mucins, porcine gastric mucin (10 mg/mL) and human intestinal mucin (10 mg/mL) were treated with either control GMM, *A. mucolyticum* conditioned medium or heat-inactivated conditioned medium (30 min, 98°C). Following incubation (18 h), the mucins were separated using SDS-PAGE and then either stained in-gel with a Periodic acid-Schiff stain (PAS) or electroblotted onto a nitrocellulose membrane and detected with a SNA lectin or an antibody against human MUC2. The PAS-based detection, which detects polysaccharides, clearly demonstrated that both the porcine gastric mucin (Figure 5a) and human intestinal mucin (Figure 5b) were almost entirely degraded by the *A. mucolyticum* conditioned medium. Heat-inactivation completely abolished the mucin degradation activity. When detecting sialic acids at terminal positions in the glycan chain by probing with the lectin SNA, a clear reduction in signal upon treatment with conditioned medium was observed (Figure 5a-b). Interestingly, human intestinal mucin, which according to the PAS stain barely entered the gel, appeared as a MUC2-positive smear after treatment with the active conditioned medium. The smear pattern indicates degradation of MUC2 and an enhanced ability to be separated by SDS-PAGE.

**Figure 6.**
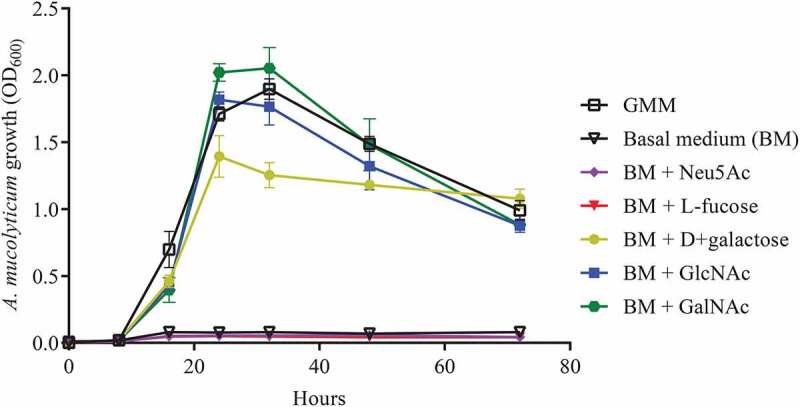
***A. mucolyticum* uses mucin *O-*glycan monosaccharides as a substrate for growth**. *A. mucolyticum* growth was assessed over a 72 h period by measuring optical density at 600 nm (OD_600_). Bacteria were grown in Gut Microbiota Medium (GMM), basal medium (BM) or basal medium supplemented with individually added monosaccharides (20 mM). Graphs depict the mean values ± SD acquired from three independent experiments

To assess whether mucin *O-*glycans products could be used as a substrate for bacterial growth, *A. mucolyticum* was cultured in a basal medium supplemented with 20 mM of the individual monosaccharides that are typically found within *O-*glycan chains: sialic acid, fucose, galactose, GlcNAc and GalNAc (Figure 6). The addition of sialic acid or fucose to basal medium did not support growth. In contrast, galactose, GlcNAc and GalNAc did support growth equal to growth levels observed in complete GMM. As sialic acid and fucose normally cap the *O-*glycans and protect the glycan chain from hydrolysis, this finding supports a model in which *A. mucolyticum* produces sialidases and fucosidases in order to gain access to and liberate underlying galactose, GlcNAc and GalNAc. Overall these data demonstrate that *A. mucolyticum* can effectively degrade mucin *O*-glycans by secreting a wide repertoire of mucin degrading enzymes after which it can use the liberated galactose, GlcNAc and GalNAc as substrates for growth.

## Discussion

While the intestinal mucus layer is designed as a barrier against bacterial invasion some bacteria have adapted to living in the mucosal niche by feeding on mucin glycans. Through close proximity to intestinal epithelial cells these bacteria can have a dominant effect on host physiology and intestinal immune responses. Here we provide evidence that the recently classified bacterium *Allobaculum mucolyticum* produces a large repertoire of mucin *O*-glycan targeting enzymes that allow it to degrade mucins and forage on host-derived mucin *O-*glycans.

A search of CAZymes encoded in the *A. mucolyticum* genome revealed an array of 60 putative CAZymes of which many can target mucin *O-*glycans. With multiple glycoside hydrolases predicted to target similar glycosidic linkages, there appears to be a considerable redundancy in the *A. mucolyticum* CAZyme repertoire. For example, there are eight putative β-galactosidases and eight different hexosaminidases. Closer inspection of the proteins revealed that both the domain architecture and organization (Figure 2) and levels of protein production (Figure 3) are markedly different between different CAZymes that are predicted to target the same linkage. This redundancy may be explained by placing it in the perspective of the incredible diversity of glycans and glycan-linkages present in intestinal mucosal niche. Mucin glycans can be branched and contain various structures and modifications, which can greatly affect enzyme-substrate interactions. Having a diverse repertoire of CAZymes therefore allows *A. mucolyticum* to utilize a wide range of glycan substrates.

Large repertoires of CAZymes are also found in the genomes of other well-known mucolytic bacteria such as *Akkermansia muciniphila, Ruminococcus gnavus, Ruminococcus torques* and several *Bifidobacteria* and *Bacteroides* species, such as *Bacteroides thetaiotamicron*.^6^ Glycan generalists such as *B. fragilis* and *B. thetaiotamicron* contain a very extensive repertoire of CAZymes, which allows utilization of a wide variety of substrates from both dietary- and host-derived sources. The total repertoire of CAZymes encoded within the *A. mucolyticum* genome is not as extensive as that of certain *Bacteroides* species, but *A. mucolyticum* seems to be highly specialized to target mucin *O-*glycans, with more than nearly half of its glycoside hydrolases directed to such glycans. This is comparable to the CAZyme repertoire detected in the mucin specialist *A. muciniphila* (Table S1). Even though the *A. mucolyticum* CAZyme repertoire is largely directed toward mucin glycans, *A. mucolyticum* can also use dietary-derived polysaccharide inulin as a substrate for growth (Figure S1). This gives the bacterium the ability to switch between dietary- and host-derived glycans and adapt to fluctuations in nutrient availability as encountered in the dynamic environment of the gastrointestinal tract.

The plasticity of the CAZyme expression in response to changes in the nutritional environment is also evident from our proteomics analysis. This reveals that *A. mucolyticum* produces a limited set of CAZymes in basal medium, that contains no source of glycans. Under this condition, which allows for minimal growth, four CAZymes were found to be significantly enriched in the conditioned medium as compared to the whole cell lysate. The four enzymes (Allo_589, 898, 1556 & 2010) are a putative α-fucosidase, a carbohydrate esterase, a β-galactosidase and a N-acetyl-β-galactosaminidase, respectively. Although the protein function and exact specificity of CAZymes are difficult to predict based on their amino acid sequence alone, the putative annotation suggests that under nutrient limited conditions *A. mucolyticum* is able to hydrolyze several host-associated glycans. For example, fucose is known to be abundant on mucin glycans, but also on glycans such as the Lewis antigens, which are known to play a role in regulating inflammation.^21^ The *N*-acetyl-β-galactosaminidase (GH123) could be involved in the degradation of glycospingholipids on the membranes of the intestinal epithelial cells.^22^

When basal medium was supplemented with either mucin or the GMM carbohydrates glucose, fructose and the α- and β-glucans maltose and cellobiose, production and secretion of CAZymes – in particular *O-*glycan targeting CAZymes – by *A. mucolyticum* was markedly increased. These results indicate that *A. mucolyticum* requires the presence of environmental glycans as stimuli to induce expression and secretion of the repertoire of glycosidases. Such regulatory mechanisms are also known from other bacteria that forage on mucin glycans.^4^ While we have not yet determined in detail the individual components responsible for the induction of the CAZymes, the fact that both mucin and the combination of glucose, fructose, maltose and cellobiose are sufficient for glycosidase expression suggests that a similar glycosidase program is initiated by glycans of both host and dietary origin.

The annotation and detection of secreted *O*-glycan targeting CAZymes did not in all cases translate to the detection of the predicted enzymatic activity. For example, while three putative fucosidases were identified within the conditioned medium of *A. mucolyticum* by mass spectrometry, no detectable fucosidase activity was observed in the conditioned medium using the monovalent 4-MU-fucose fluorescent substrate even though the validity of the assay was confirmed with a commercial fucosidase. Nor could fucosidase activity be detected using a novel activity-based probe. Although it is possible that all three fucosidases were annotated incorrectly by the dbCAN2 pipeline, the lack of detectable fucosidase activity is perhaps more likely explained by an incompatibility between the substrate specificity of the *A. mucolyticum* fucosidases and the monovalent substrates used in this study, as monovalent substrates are structurally different than the complex plant- and host-derived glycans found in the intestine.

In contrast to the lack of detectable levels of fucosidase activity, we were able to detect *N*-acetyl-α-galactosaminidase activity, which was not expected on basis of the CAZyme annotation (Table 1). This might indicate that *A. mucolyticum* is capable of hydrolyzing the α-linked GalNAc moiety that makes up the core of *O-*glycans, thereby enabling the complete degradation of mucin glycans. This hypothesis is supported by the finding that *A. mucolyticum* can effectively utilize GalNAc for growth (Figure 6). The enzyme(s) responsible for the *N*-acetyl-α-galactosaminidase activity remain to be identified.

Crost *et al*.^23^ have previously demonstrated that the ability of *R. gnavus* to grow on PGM as a sole carbon source is strain-dependent, and was due to the presence of sialidases and fucosidases. These results highlight the necessity for both types of glycosidases to remove the terminal sialic acid and fucose residues in order to allow degradation and subsequent utilization of the underlying glycans. *A. mucolyticum* produces multiple sialidases and fucosidases that are suspected to be essential to access mucin glycans for growth. Media supplemented with sialic acid or fucose, however, were not able to support growth (Figure 6), which is in agreement with the absence of core genes in the *A. mucolyticum* genome normally found in canonical gene clusters for sialic acid or fucose catabolism. Interestingly, a curated blast search of the genome does show the presence of several genes predicted to be involved in the uptake of sialic acid, like genes encoding for sialic acid TRAP transporter SiaQ and sialic acid-binding protein SiaP (Allo_00009 and Allo_00087, respectively).^24^ Furthermore, there are several putative polysialyltransferases (e.g. Allo_01518 and Allo_02009) with homology to proteins from *Neisseria meningitidis* known to play a role the sialylation of lipooligosaccharide (LOS). Sialylation of the bacterial LOS or capsule glycans is known to contribute to host colonization and bacterial immune evasion.^25,26^ When *A. mucolyticum* does not directly use host glycan-liberated sialic acid or fucose, they could still have a major impact on the ecology of the mucosal niche and host health as free sialic acid and fucose has been shown to boost the expansion of enteric pathogens like *Salmonella typhimurium, Clostridium difficile, Campylobacter jejuni* and *Escherichia coli* under certain conditions.^27–30^ In addition to this, removal of sialic acid from mucin glycans was shown to affect rotavirus adhesion and infection.^31,32^ Whether *A. mucolyticum* decorates its surface glycans with endogenous sialic acid or fucose, or affects the microbiota composition indirectly by releasing them into the environment remains to be investigated.

Increased bacterial penetration and degradation of the intestinal mucus layer are major features of IBD. Understanding the mechanisms that drive these processes may thus be key to treating or preventing disease. It has been reported that a diet deprived of dietary fiber enhances the expression of mucin targeting CAZymes of the gut microbiota and promotes the expansion of mucus degrading bacteria such as *A. muciniphila* and *Bacteroides caccae*.^33^ As mucin also seems to be an important nutrient source for *A. mucolyticum*, it may be that a similar expansion might be observed for this bacterium. Bacterial mucus degradation may cause erosion of the mucus layer and an increased susceptibility to lethal colitis by the enteric pathogen *Citrobacter rodentium*, as was shown in mice.^33^ This finding highlights the delicate balance between the microbiota, an intact mucus layer and invading pathogens.

Mucus layer degradation, as observed in IBD patients, is linked to an increased prevalence mucosa-associated mucolytic bacteria, like *R. gnavus* and *R. torques*.^34^ Since *A. mucolyticum* was isolated from a patient with ulcerative colitis and, as described here, is able to effectively forage on mucins, it is likely that it operates in the same niche as other mucolytic bacteria. It would therefore be of great interest to investigate the interactions with other known mucus-degrading bacteria and to investigate to what extent the mucolytic capacities of *A. mucolyticum* can contribute to the pathogenesis of IBD.

## Materials and methods

### General

All commercial chemicals were purchased from Sigma-Aldrich unless stated otherwise. Data visualization was performed using Adobe Illustrator 2021, GraphPad Prism 7, and R.

### Carbohydrate-active enzyme (CAZyme) annotation

CAZymes within the Prokka-annotated genome of *A. mucolyticum* (Genbank accession number: JAHUZH010000000) and *A. muciniphila* ATCC BAA-835 (NCBI Reference Sequence: NC_010655.1) were annotated using the dbCAN2 metaserver (http://bcb.unl.edu/dbCAN2/) in February 2021.^20,35^ CAZymes identified by at least two out of three tools were considered for further analysis and are displayed in Figure 1. The putative mucin-targeting glycoside hydrolases, as displayed in Table 1, Figure 2, and supplementary STable 1, were selected based on the mucin targeting CAZyme classes reported by Tailford *et al*.^6^

### Mucin and mucin O-glycan purification

Porcine gastric mucin (PGM type III, Sigma–Aldrich) was purified by ethanol precipitation as described previously by Ottman *et al*.^36^ with minor modifications. Briefly, 10 g of mucin was dissolved in 500 ml 2X PBS with pH 7.8 (NaCl: 274 mM, KCl: 5.4 mM, Na_2_HPO_4_: 20 mM, KH_2_PO_4_: 3.6 mM) and 100 µL of toluene for 24 h at 4°C, under continuous stirring. After the first hour, the pH was adjusted to 7.2 with 2 M NaOH. After 24 h, the solution was centrifuged at 10,000 x *g* for 20 min at 4°C, after which the supernatant containing the dissolved mucin was collected, cooled to 0°C and ice-cold (0°C) ethanol was added to a final concentration of 60% (vol/vol). The resulting precipitate was dissolved in 0.1 M NaCl and precipitated again with ice-cold ethanol to 60% (vol/vol). The precipitate was then washed with 100% ice-cold ethanol and dissolved in distilled water (~180 mL). Thereafter, the mucin solution was dialyzed against 5 L of distilled water for 24 h at 4°C, lyophilized, resuspended in Milli-Q ultrapure water, and sterilized by autoclaving (15 min at 121°C).

Porcine gastric mucin *O*-glycans were purified according to a protocol by Desai *et al*.^33^ In short, PGM (Type III, Sigma-Aldrich) was suspended at 2.5% w/v in 100 mM Tris (pH 7.4) and autoclaved immediately (15 min at 121°C). Proteinase K (Sigma) was added to a final concentration of 100 µg/mL and incubated for 16 h at 55°C with slow shaking. The proteolyzed solution was centrifuged at 21,000 x *g* for 30 min at 4°C to remove insoluble material. Next, NaOH and NaBH_4_ were added to final concentration of 0.1 M and 1 M, respectively. This solution was incubated for 18 h at 65°C to promote selective release of mucin *O*-glycans from mucin glycopeptides by alkaline β-elimination. The solution was neutralized to pH 7.0 with 2 M HCl, centrifuged at 21,000 x *g* for 30 min at 4°C, and filtered with a 0.2 µm filter to remove remaining insoluble material. After extensive dialysis (4 x 4 h with a 100X volume of Milli-Q Ultrapure water, 1 kDa MWCO dialysis tube), the dialyzed mucin *O*-glycan solution was lyophilized and resuspended in Milli-Q Ultrapure water at desired concentrations.

Human small intestinal mucins were harvested from the urine (~100 mL per batch) of a patient with an orthotopic neobladder reconstruction. Crude mucin was collected by centrifugation (30,000 x g, 4°C, 30 min) and pellets were washed twice with PBS. Washed mucin was purified further using ethanol precipitation, as described for PGM, and redissolved in 5 mL Milli-Q Ultrapure water. Instead of lyophilization, the mucin was directly transferred to a 100 kDa MWCO Amicon Ultra filter (Thermo Fisher Scientific), washed twice with 5 mL Milli-Q and concentrated. Protein concentration was determined using a NanoDrop One UV-Vis Spectrophotometer (Thermo Fisher Scientific) and set to 10 mg/mL in Milli-Q Ultrapure water.

### Bacterial culture conditions

*A. mucolyticum* was routinely cultured at 37°C under anaerobic conditions in a vinyl anaerobic chamber (Coy Labs) with the following gas mix: 85% N_2_, 10% CO_2_ and 5% H_2_. Gut Microbiota Medium (GMM) (Table S2), was the default growth medium and is essentially an enriched Gut Microbiota Medium, as described by Goodman *et al*.^37^ that lacks isovaleric acid. The basal medium (BM) is GMM lacking glucose, fructose, maltose and cellobiose. Prior to use for bacterial growth, all media were prereduced in the anaerobic chamber for at least 12 h.

### Bacterial growth curves

Gut Microbiota Medium and basal medium were prepared according to recipe in described in Table S2. All media supplements were prepared in Milli-Q Ultrapure water and, if required, solutions were brought to neutral pH using 1 M NaOH. Supplemented media were prepared with supplements at following final concentrations: D+ glucose (2 mg/ml, Sigma), D-fructose (1 mg/mL, Sigma), D+ maltose (1 mg/ml, Sigma), D+ cellobiose (1 mg/ml, Sigma), N-Acetylneuraminic acid (20 mM, Carbosynth), L-Fucose (20 mM, Sigma), D+ Galactose (20 mM, Mikrobiologie), N-acetyl-D-glucosamine (20 mM, Sigma), N-acetyl-D-galactosamine (20 mM, Carbosynth), inulin (10 mg/mL, Sigma), purified porcine gastric mucin (PGM, 0,5% w/vol) or purified PGM *O*-glycans (10 mg/mL). All solutions, except PGM solution, were filter-sterilized and prereduced in the anaerobic chamber prior to use

*A. mucolyticum* was grown on GMM agar plates for 72 h at 37°C under anaerobic conditions. Static liquid starter cultures were inoculated with three separate bacterial colonies each and grown for 16 h at 37°C under anaerobic conditions. Bacterial growth was assessed by measuring absorbance (600 nm). Bacteria were collected by centrifugation (3,000 x g, 5 min), gently washed twice and resuspended in 2X basal medium. Washed bacteria were diluted to the desired OD and combined with an equal volume of MilliQ Ultrapure water or medium supplements to a total volume of 200 µL per well with a final starting OD of 0.01. For each time point (0, 8, 16, 24, 32, 48 and 72 h) a 96-Well Multiwell Plate (Corning Costar) was used with duplicate wells per condition. At each time point one plate was removed from the anaerobic chamber and final absorbance values (600 nm) were recorded using a FLUOstar Omega plate reader (BMG Labtech). Values were corrected for background levels in respective negative controls. Experiments were performed in triplicates, using three biologically independent *A. mucolyticum* starter cultures.

### Bacterial conditioned media and whole cell lysate preparation

Bacterial conditioned media and whole-cell lysates for mass spectrometric analysis were harvested from static liquid *A. mucolyticum* cultures that were grown in Gut Microbiota Medium, basal medium or basal medium supplemented with 0.5% PGM for 24 h at 37°C under anaerobic conditions. For each condition, 4 mL cultures were inoculated from three independent starter cultures and grown as described above. After 24 h, bacteria were harvested by centrifugation at 15,000 x *g* for 2 min, washed twice with 2 mL of PBS and resuspended in 4 mL of ice-cold lysis buffer (PBS containing cOmplete EDTA-free protease inhibitor cocktail (Roche)). The conditioned media were collected and filter-sterilized using 0.2 µm filter. Bacteria were lysed by sonication (3 x 10 seconds on ice). Large bacterial fragments were removed by centrifugation at 15,000 x *g* for 1 min, after which cleared lysate was collected. To remove possible media components all bacterial cell lysates and conditioned media were transferred to a 5 kDa MWCO filter (Pierce concentrator, PES, 5 K MWCO, 6 ml, Thermo Fisher Scientific), washed with 5 mL of Milli-Q and concentrated to 1 mL.

The conditioned media used for glycosidase enzymatic activity assays were collected from three independent static liquid *A. mucolyticum* cultures grown in the Gut Microbiota Medium for 48 h at 37°C under anaerobic conditions. The conditioned media were filter-sterilized using 0.2 µm filter and stored at −20°C prior to use. The 100X concentrated conditioned media used for fucosidase assays were obtained using a 10 kDa MWCO Amicon Ultra filter (Thermo Fisher Scientific).

### Mass spectrometry sample preparation

Whole cell lysates and conditioned media were processed using Filter Aided Sample Preparation (FASP), as described by Wiśniewski *et al*.^38^ In brief, samples were denatured at 95°C in the presence of DTT, mixed with 8 M urea and loaded onto Centrifugal Filters (Microcon, cat. no MRCF0R030). This was followed by two washes with 8 M urea, treatment with 0.05 M iodoacetamide in 8 M urea, and three 8 M urea washes. After washing out the urea with three washes with 0.05 M ammonium bicarbonate, samples were digested overnight with trypsin at 37°C. The next day, samples were acidified and desalted using Stagetips.^39^ Half of each of the digested samples was injected into an LTQ-Orbitrap QExactive mass spectrometer (Thermo Fisher Scientific) and measured using a 120-minute gradient.

### Mass spectrometry analyses

Thermo Raw files were analyzed using MaxQuant versions 1.5.1.0 using default parameters, with the inclusion of the match between runs and IBAQ features.^40,41^ Initial analyses were performed in Perseus.^42^ Proteins flagged as contaminants, reverse or only identified by site were filtered out. Triplicates were grouped and only proteins reproducibly quantified in at least one of the sets of triplicates were retained. Missing values were imputed using default parameters. Differential proteins were determined using a t-test with adjustment for multiple testing (FDR < 0.05). To call proteins enriched in the secreted fractions, they additionally required at least a 10-fold higher LFQ value compared to the whole cell lysates. Data visualization and downstream processing was performed in R.

### Glycosidase activity assays

*A. mucolyticum* conditioned media used for enzymatic assays were collected as described above. The following substrates were used: 4-MU-Neu5Ac (Cayman), 4-MU-Fucose (Carbosynth), 4-MU-GlcNAc (Carbosynth), 4-MU-GalNAc (Carbosynth), and ortho-Nitrophenyl-β-galactoside (ONPG, Sigma). Stocks of the 4-MU linked substrates were diluted to 200 µM in Milli-Q Ultrapure water and ONPG was diluted to 1 mg/mL in Milli-Q Ultrapure water, after which 50 µl of diluted substrates were added to a 96-Well Multiwell flat-bottom Plate (Corning Costar). Next, 50 µL of either the Gut Microbiota Medium (negative control), conditioned medium or heat-inactivated conditioned medium (98°C for 30 min) were added in duplicates. Plates were incubated for 2 h at 37°C shielded from light and transferred to a FLUOstar Omega plate reader (BMG labtech). For the 4-MU linked substrates, signal was detected using a 340 ex./460 em. filter set. For the ONPG substrate, absorbance was measured at a wavelength of 420 nm.

Chemoselective labeling of fucosidases using an activity-based probe was performed as described by Luijkx and Henselijn *et al*.^43^

### Detection of mucin degradation by Periodic acid-Schiff (PAS) stain and western blotting

PGM or human mucin (10 mg/mL in Milli-Q Ultrapure water) were combined at a 1:1 ratio with medium controls or the same *A. mucolyticum* conditioned medium that was used for the glycosidase activity assays. This mixture was incubated for 16 h at 37°C after which samples were mixed with one volume of 3x Laemmli sample buffer and boiled for 5 min. Subsequently 25 µL per sample was loaded onto a gel and separated using SDS-PAGE. For SDS-PAGE separation and immunoblotting of large mucin proteins, a Boric acid-Tris gel system was used, as previously described by Li *et al*,^44^ with minor modifications. A 5% acrylamide gel (5% Acryl/Bis acryl solution, Bio-Rad 161–0144; 26% 1.5 M Tris pH 8.8; 0.1% SDS; 0.1% ammonium persulfate; 0.1% TEMED) was prepared in a Mini Protean II chamber (Bio-Rad) using 1.5 mm spacer plates. Gels were run in Boric acid-Tris buffer (192 mM Boric acid; 1 mM EDTA; 0.1% SDS, set to pH 7.6 with Tris) at 25 mA per gel for 3 h. To detect glycoproteins, gels were stained using a Periodic acid-Schiff stain (Pierce glycoprotein staining kit, Thermo Fisher Scientific). For detection of MUC2 and sialic acids, mucins were separated as described above and transferred onto nitrocellulose membranes using a wet transfer system with transfer buffer (25 mM Tris; 192 mM glycine; 20% methanol,) for 3 h at 90 V at 4°C. Subsequently, membranes were blocked with 5% bovine serum albumin (BSA, Sigma-Aldrich) in TSMT buffer (20 mM Tris, 150 mM NaCl, 1 mM CaCl_2_, 2 mM MgCl_2_, 0.1% Tween 20, adjusted to pH 7 with HCl) overnight at 4°C and then incubated with an anti-human MUC2 rabbit serum (a gift from Dr. Karin Strijbis) at a 1:200 dilution in TSMT containing 1% BSA, or with biotinylated SNA lectin (2 mg/mL stock, Vector Labs) at a dilution of 1:1000 in TSMT containing 1% BSA. After incubation for 1 h at room temperature, blots were washed 4 times with an excess of TSMT and incubated with α-rabbit IgG (A4914, 1:10,000 dilution, Sigma-Aldrich) in TSMT containing 1% BSA for the detection of MUC2, or Streptavidin-HRPO (1 mg/ml stock, 1:10,000 dilution, Jackson ImmunoResearch) in TSMT containing 1% BSA for the detection of sialic acids. Blots were developed with the Clarity Western ECL kit (Bio-Rad) and imaged in a Gel-Doc system (Bio-Rad).

## Supplementary Material

Supplemental Material

## Data Availability

The mass spectrometry proteomics data have been deposited to the ProteomeXchange Consortium via the PRIDE partner repository with the dataset identifier PXD024919.^45^ Data can be accessed via https://www.ebi.ac.uk/pride/ using dataset identifier above. The *A. mucolyticum* Whole Genome Shotgun project has been deposited at DDBJ/ENA/GenBank under the accession JAHUZH000000000. The version described in this paper is version JAHUZH010000000.
