# Supplementary material for: Identification of Allobaculum mucolyticum as a novel human intestinal mucin degrader
Source: Gut Microbes. 2021 Aug 30;13(1):1966278. doi: 10.1080/19490976.2021.1966278 (PMC8409761; doi:10.1080/19490976.2021.1966278)
Supplement: Supplemental Material [file KGMI_A_1966278_SM7882.zip › Supplementary information/Supplementary legends.docx]

**Table S1. Comparison of number of putative mucin *O*-glycan targeting CAZymes of *A. mucolyticum* and *A. muciniphila* ATCC BAA-835**

**Table S2. Recipe for enriched Gut Microbiota Medium (GMM)**

**Fig. S1. *A. mucolyticum* can use inulin as substrate for growth**. *A. mucolyticum* growth was assessed over a 72 h period by measuring optical density at 600nm (OD_600_). Bacteria were grown in Gut Microbiota Medium (GMM), basal medium (BM) or basal medium supplemented with inulin (10mg/mL). Graphs depict the mean values ± SD acquired from three independent experiments.

**Fig. S2. Mass spectrometry analysis of *A. mucolyticum* proteome.** Conditioned media from 24 h old cultures in basal medium (BM), basal medium + PGM and Gut Microbiota Medium (GMM) and a whole cell lysate (WCL) from the GMM culture were subjected to mass spectrometry analysis, with three biologically independent replicates per condition. (A**)** The three scatter plots show the log2 LFQ from whole cell lysate of the GMM culture (X-axis) and the secretomes from the three different media (Y-axis). The colored dots indicate the proteins with >10 fold enrichment in the secretome versus GMM WCL. (B) The red/blue heat map displays the proteins and their relative enrichment in the three different secretomes compared to the GMM WCL. Significance (depicted in the brown heatmap) was calculated using a t-test and indicates a >10 fold enrichment in the respective secretome versus GMM WCL with a FDR < 0.05.

**Fig. S3. Mass spectrometry analysis of *A. mucolyticum* CAZymes.** Conditioned media from 24 h old cultures in basal medium (BM), basal medium + PGM and Gut Microbiota Medium (GMM) and a whole cell lysate (WCL) from the GMM culture were subjected to mass spectrometry analysis, with three biologically independent replicates per condition. (A**)** The three scatter plots show the log2 LFQ from whole cell lysate of the GMM culture (X-axis) and the secretomes from the three different media (Y-axis). The colored dots indicate the CAZymes. (B) The red/blue heat map displays all the putative CAZymes and their relative enrichment in the three different secretomes compared to the GMM WCL. Significance (depicted in the brown heatmap) was calculated using a t-test and indicates a >10 fold enrichment in the respective secretome versus GMM WCL with a FDR < 0.05.
