# Supplementary figures and images for: Identification of Allobaculum mucolyticum as a novel human intestinal mucin degrader
Source: Gut Microbes. 2021 Aug 30;13(1):1966278. doi: 10.1080/19490976.2021.1966278 (PMC8409761; doi:10.1080/19490976.2021.1966278)

Figure S1

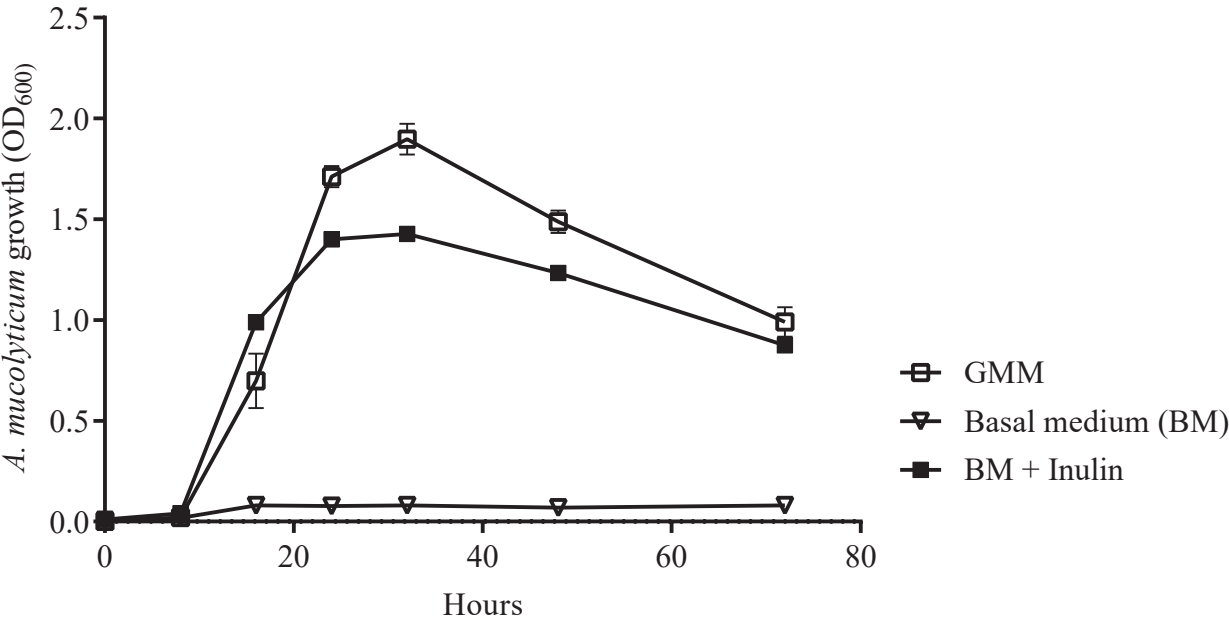

Figure S2

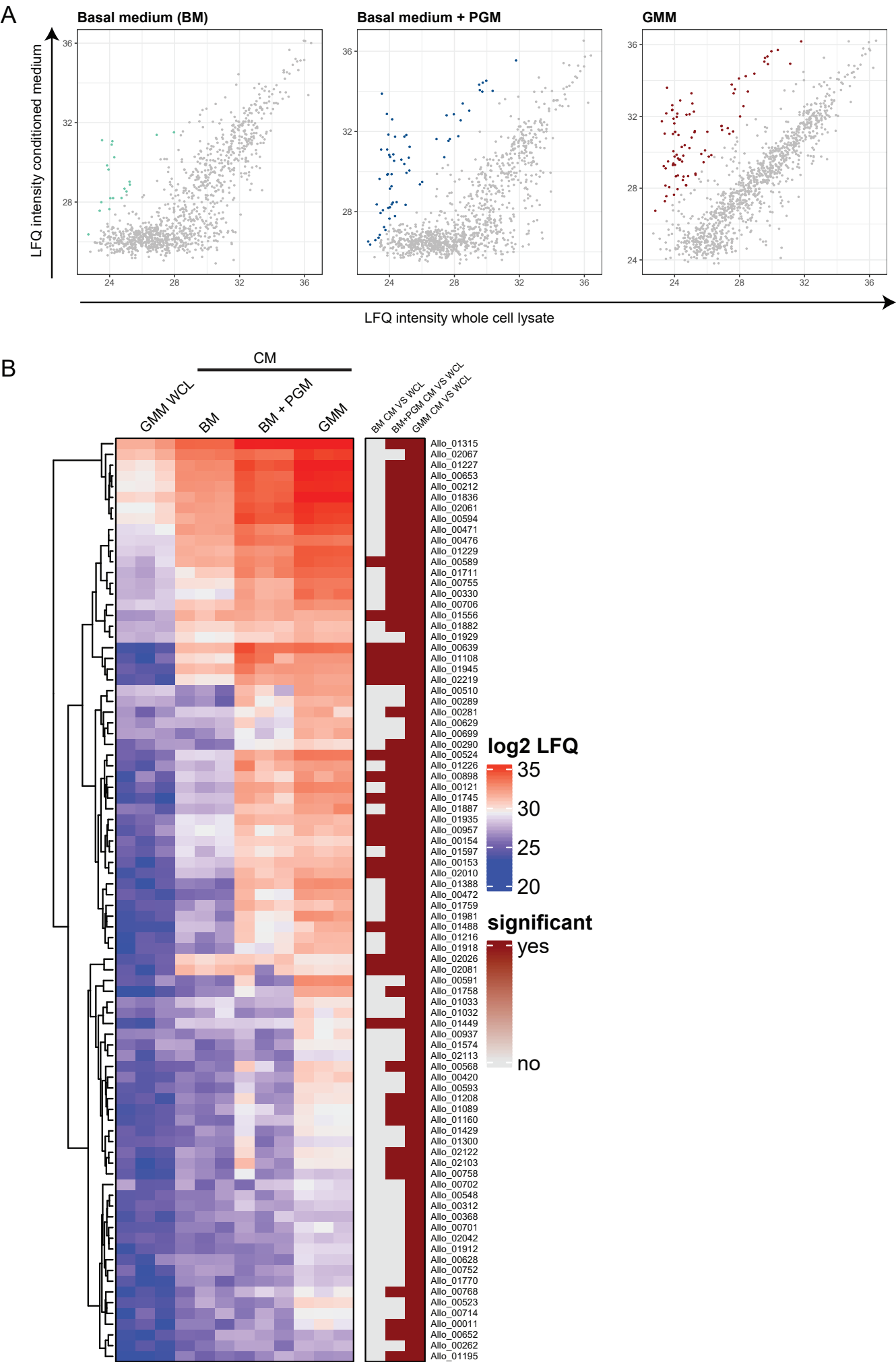

Figure S3

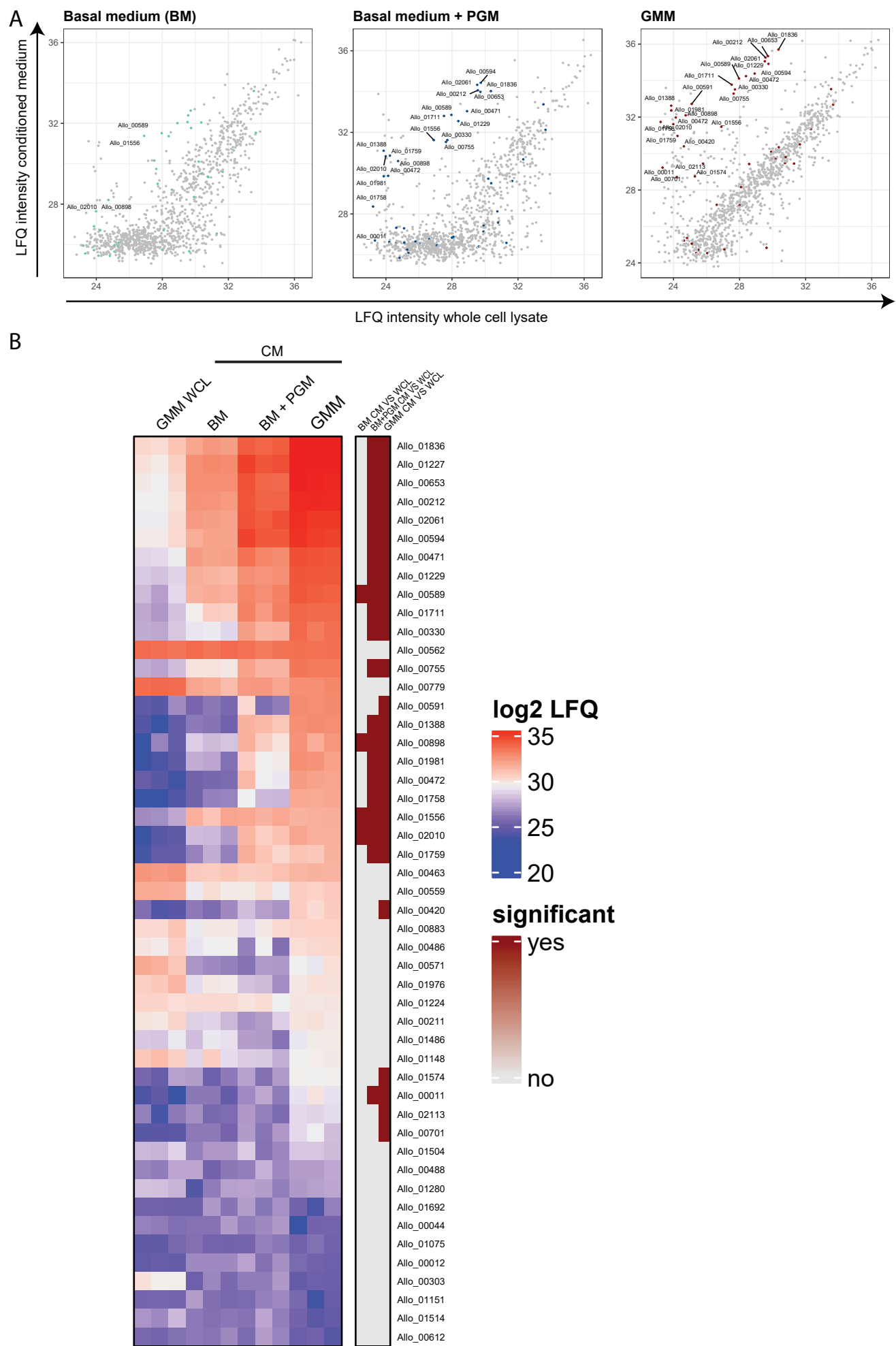

Supplement: Supplemental Material [file KGMI_A_1966278_SM7882.zip › Supplementary information/Supplementary Figures_revised.pdf]
